# A Proof-of-Concept Ecological Momentary Assessment Study of Day-Level Dynamics in Value-Based Decision-Making in Opioid Addiction

**DOI:** 10.3389/fpsyt.2022.817979

**Published:** 2022-05-17

**Authors:** Emmanuel E. Alvarez, Sahar Hafezi, Darla Bonagura, Evan M. Kleiman, Anna B. Konova

**Affiliations:** ^1^Department of Neuroscience, Robert Wood Johnson Medical School, Rutgers University–New Brunswick, Piscataway, NJ, United States; ^2^Department of Psychiatry, Brain Health Institute, University Behavioral Health Care, Rutgers University–New Brunswick, Piscataway, NJ, United States; ^3^Department of Psychology, University of Tennessee, Knoxville, Knoxville, TN, United States; ^4^Department of Psychology, Rutgers University–New Brunswick, Piscataway, NJ, United States

**Keywords:** ecological moment assessment, decision-making, risk tolerance, impulsive choice, state-dependence, mood, substance use disorder

## Abstract

**Background:**

Drug addiction is thought to be characterized by risky and impulsive behavior despite harmful consequences. Whether these aspects of value-based decision-making in people with addiction are stable and trait-like, and the degree to which they vary within-person and are sensitive to changes in psychological state, remains unknown. In this pilot study, we examined the feasibility of distinguishing these state- vs. trait-like components by probing day-level dynamics of risk and time preferences in patients with opioid use disorder (OUD) as they engaged with their natural environment.

**Methods:**

Twenty-three individuals with OUD receiving outpatient treatment (40% female; *M* = 45.67 [*SD* = 13.16] years of age) and twenty-one matched healthy community controls (47% female; *M* = 49.67 [*SD* = 14.38] years of age) participated in a 28-day smartphone-based ecological momentary assessment study (1085 person days; *M* = 24.66, *SD* = 5.84). Random prompts administered daily assessed subjects’ psychological state (e.g., mood) and economic preferences for real delayed and risky monetary rewards.

**Results:**

Subjects demonstrated dynamic decision-making preferences, with 40–53% of the variation in known risk and ambiguity tolerance, and 67% in discounting, attributable to between-person vs. within-person (day-to-day) differences. We found that changes in psychological state were related to changes in risk preferences, with patients preferring riskier offers on days they reported being in a better mood but no differences between groups in aggregate level behavior. By contrast, temporal discounting was increased overall in patients compared to controls and was unrelated to global mood. The study was well-tolerated, but compliance rates were moderate and lower in patients.

**Conclusion:**

Our data support the idea that decision-making preferences in drug addiction exhibit substantial within-person variability and that this variability can be well-captured using remote data collection methods. Preliminary findings suggested that aspects of decision-making related to consideration of risk may be more sensitive to within-person change in global psychological state while those related to consideration of delay to reward, despite also being somewhat variable, stably differ from healthy levels. Identifying the cognitive factors that contribute to opioid use risk in a “real-world” setting may be important for identifying unique, time-sensitive targets for intervention.

## Introduction

The opioid epidemic is a public health crisis with alarming personal and societal costs, with opioid use and overdose accounting for over 1,00,000 deaths annually and an estimated $504 billion in related costs in the United States (1, 2). Given the harmful consequences of chronic drug use, drug addiction is thought to affect normative decision-making and valuation mechanisms, in particular those requiring integration of information about risk and time (delay) into the choice process (3–5). However, whether putative biases in decisions involving risk and delay constitute a stable feature of people with addiction or are a flexible reflection of the current state of an individual, remains poorly understood. Determining the stability, or conversely dynamic nature, of these cognitive processes is of particular interest as this may inform our understanding of the mechanisms that confer risk for and/or maintain addiction, as well as reveal better targets for intervention.

Value-based decision-making refers to the process by which we make decisions based on the subjective values of available choice options with the goal of maximizing reward and minimizing unfavorable outcomes (6). While there are many individual difference factors that can influence subjective value computations, two that have received considerable attention in the addiction literature are temporal discounting and risk tolerance (7, 8). *Temporal discounting*, also known as delay discounting, refers to how people devalue rewards received in the future compared to those received at a sooner time, with individuals largely preferring smaller, more immediate rewards compared to larger, delayed rewards. *Risk tolerance* broadly refers to a person’s propensity to approach or avoid risk and uncertainty. *Known risk* involves knowledge about the explicit odds of a given outcome, while *ambiguous (unknown) risk* refers to conditions where the exact odds are unknown or cannot be estimated. Cross-sectional research has revealed consistent differences in these decision-making preferences across individuals who use substances when compared to healthy controls. For instance, people with substance use disorders, regardless of treatment status or preferred substance, consistently exhibit excessive discounting behavior (7, 9–12). Similarly, additional research has identified increased risk tolerance in patients with substance use disorder (8, 13–18) although effect sizes here tend to be smaller, and the type of risk probed (known vs. ambiguous risk) is not always distinguished.

While this literature suggests the presence of aggregate—potentially *trait-level*—differences from healthy individuals in value-based decision-making, addiction is a chronic disease characterized by a cyclic pattern of preoccupation-anticipation, drug use, and abstinence/withdrawal (19). The transition between these clinically relevant phases may be subject to substantial within-person change that may be missed in cross-sectional research. Dynamic changes in value-based decision-making have, however, rarely been examined in people with addiction, and typically not by probing more than one influential factor at a time (e.g., different forms of risk) or by acquiring more than a handful of measurements per subject (20). Computational parsing of the latent drivers of decision-making are also rare beyond discounting but may provide a more nuanced depiction of how distinct aspects of decision-making contribute to substance use (21). A combined approach addressing these limitations is needed to identify which cognitive decision processes might reflect changes at short timescales (e.g., days, weeks, or months) that can guide ongoing treatment or prognosis, and which might primarily reflect stable features of the disorder that could inform early prevention efforts.

We recently studied a constrained set of preferences in response to known risk and ambiguity as captured by validated economic decision-making tasks in a lab-based setting (22). We found that treatment-engaged individuals with opioid use disorder (OUD) displayed, at the roughly week-to-week timescale, variable tolerance for ambiguity that was linearly related to ongoing vulnerability for drug reuse over 6 months. These data suggested that some decision-making preferences, such as ambiguity tolerance, may be more dynamic than previously thought in addiction, consistent with emerging research pointing to sizable variation in risky decision-making as captured by other measures such as the Balloon analog Risk Task (23), and by temporal discounting (24), in individuals who use substances. These studies also suggest the cognitive processes captured by these measures may more closely reflect the current psychological state of the individual, such as good or poor mood (25–28) or symptom exacerbation/instability (29–32), than a stable feature of the disorder. However, this prior work has not investigated risk tolerance and temporal discounting together and in most cases has not had the necessary temporal resolution to assess the relative contribution of within-person (state-dependent) vs. between-person (trait-like) effects in these decision-making preferences.

To study the temporal patterns of decision-making in addiction, we employed a 28-day ecological momentary assessment (EMA) protocol in a population of patients with OUD receiving outpatient treatment. This methodology allowed us to capture fine-grained changes in decision-making not readily captured with less granular (e.g., weekly) assessments. We focused on this population given extensive prior work showing good tolerability of EMA and day-level variation in psychological states of interest (33–36). In this proof-of-concept study, we adapted to an EMA platform a battery of validated economic probes of preferences for risky and delayed monetary rewards and losses. The tasks were administered *via* a smartphone-based application daily, and we concomitantly assessed day-to-day changes in psychological state. First, we assessed the feasibility of this approach to detect meaningful decision-making behavior and changes in this behavior in this OUD population. We then tested for aggregate level differences from a comparison group of healthy community controls who completed the same procedures and for changes tied to global changes in psychological state (positive and negative mood). This allowed us to monitor how people with addiction make decisions about rewards that can be received with different costs as they engaged with their natural environment and determine the extent to which these decision-making preferences are influenced by the current psychological state of the individual and/or by individual differences.

## Materials and Methods

### Participants

**FIGURE 1:**
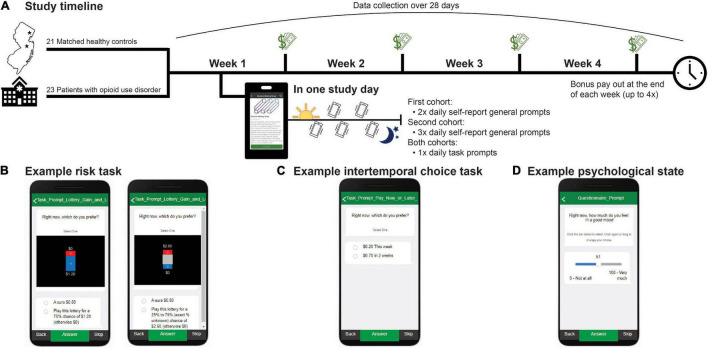
Study timeline. **(A)** Forty-four subjects were enrolled in a 28-day ecological momentary assessment study. Subjects completed self-report surveys and decision-making tasks for real risky and delayed rewards from which a financial bonus was randomly selected and paid out at the end of each week. Select example screenshots of how behavior tasks and prompts were presented within the MetricWire app for: **(B)** known risk (left) and ambiguity (right) trials; **(C)** intertemporal choice task trials; and **(D)** psychological state questions.

Treatment-engaged individuals with OUD and healthy community controls with access to internet-enabled smartphones who were >18 years of age were recruited from the northern New Jersey metropolitan area. Patients were recruited from two university-affiliated outpatient clinics that provide medications for OUD treatment. Subjects were screened and excluded for psychotic and uncontrolled affective illness that could interfere with study participation (e.g., untreated mania). Healthy controls were screened for history of mental health and substance use disorders. Forty-six individuals were enrolled across two cohorts (see section “Procedures”). The first cohort (*N* = 22; *n* = 12 with OUD) completed the 28-day EMA study between 8/05/2020 and 02/02/2021 while the second cohort (*N* = 24; *n* = 12 with OUD) was enrolled between 3/11/2021 and 09/03/2021 (Figure 1A). Two subjects (one patient and one control) requested to cease participation. Therefore, a total of 44 individuals are included in the reported analyses (*n* = 23 patients with OUD [9 female; *M* = 45.67 (*SD* = 13.16) years of age; 9% Hispanic; 57% Black/African American, 34% White, 9% Other or More than One Race]; *n* = 21 controls [10 female; *M* = 49.67 (*SD* = 14.38) years of age; 19% Hispanic; 29% Black/African American; 67% White; 4% Other or More than One Race]). There were no significant differences between the groups in age [*t*(42) = −0.96, *P* = 0.34], sex (χ^2^ = 0.32, *P* = 0.57), or race/ethnicity (χ^2^> 0.99, *P* > 0.05). Individuals with OUD reported an average of 16.0 (*SD* = 12.60) years of opioid use, including heroin and opioid analgesics (range: 1.5–52 years), and an average of 5.57 (*SD* = 4.27) treatment attempts prior to the current treatment (range: 0–14 attempts). Approximately 13% had used illicit opioids within 1 week of starting the study. Patients were predominantly stabilized with buprenorphine/naloxone (*n* = 21; 8−32 mg daily) with the remainder receiving methadone (*n* = 1; 140 mg daily) or extended-release naltrexone (*n* = 1; 380 mg monthly). The Rutgers University Institutional Review Board approved the study.

### Measures

#### Risk Task

To probe preferences for known and ambiguous risk, we adapted a previously published task (22, 37–41) (Figure 1B). On each study day, subjects were prompted to complete “gain” and “loss” versions of the task which consisted of 54 trials each (decreased from 124 trials in the original task). In the ‘gain’ condition, subjects chose between a guaranteed $0.50 and a lottery. Each lottery had two possible outcomes: $0 or *v*, with *v* equal to $0.60, $0.80, $1.20, $1.80, $2.60, $3.90, $4.40, or $6.60. In half of the trials, *v* could be received with one of three known probabilities (*p*), 25%, 50%, or 75% (i.e., risk trials). In the other half, probability information associated with winning *v* was partly occluded (i.e., ambiguity trials) resulting in one of three levels of ambiguity (*A*): 24%, 50%, or 74%. In the second condition, “loss,” all aspects of the gain condition were held constant except that all potential values, including the guaranteed option, were negative and denoted money lost from an initial endowment of $6.60. Subjects were incentivized to complete the task according to their true preferences as bonus payments, received weekly, were reflective of the subject’s actual choices (see section “Procedure”). All subjects that participated in the EMA study first received detailed instructions, passed a task comprehension quiz, and completed an extended version of the task with an experimenter, either in person in the laboratory or remotely, before initiating the EMA portion.

Trial-by-trial choice data were fit separately for each condition (gain or loss) and study day to obtain individual subject and day estimates of risk tolerance in gains, ambiguity tolerance in gains, risk tolerance in losses, and ambiguity tolerance in losses. As described previously (22, 37–41), we fit subjects’ choice data with a modified power utility model (42) (Eq. 1) that separately parametrizes known risk and ambiguity tolerance. The expected utility (*EU*) of each option offered on each trial was determined by the amount that could be gained or lost (*v*), the known probability of winning or losing (*p*), the fraction of the probability that was unknown (*A*), a subject-specific known risk tolerance parameter (α), and a subject-specific ambiguity tolerance parameter (β).


$$E\,U_{o\,p\,t\,i\,o\,n}=\left[p-\beta \,\left(\frac{A}{2}\right)\right]\,v^{\alpha }$$

To estimate α and β, we fit a single logistic function (Eq. 2) to the trial-by-trial data using maximum likelihood estimation in MATLAB version 2019b with a lower and upper bound of [0,10] for α and [−5,5] for β. The probability of choosing the lottery (*Pr*_*lottery*_) was derived from the expected utilities (from Eq. 1) of the lottery (*EU*_*lottery*_) and guaranteed (*EU*_*safe*_) options, and a third subject-specific choice stochasticity parameter (γ).


$$P\,r_{l\,o\,t\,t\,e\,r\,y}=\frac{1}{1+e^{-\gamma \,\left(E\,U_{l\,o\,t\,t\,e\,r\,y}-E\,U_{s\,a\,f\,e}\right)}}$$

In the “gain” condition, α = 1 indicates risk neutrality, α > 1 risk seeking, and α < 1 risk aversion. The reverse is true in the “loss” condition: α = 1 indicates risk neutrality, α < 1 risk seeking, and α > 1 risk aversion. In the “gain” condition, β = 0 indicates ambiguity neutrality, β < 0 ambiguity seeking, and β > 0 ambiguity aversion, and in the “loss” condition, β = 0 indicates ambiguity neutrality, β > 0 ambiguity seeking, and β < 0 ambiguity aversion. For ease of interpretation, we report risk tolerance as α-1 in gains and 1-α in losses. Similarly, we report ambiguity tolerance as –β in gains and β in losses.

#### Intertemporal Choice Task

To probe time preferences, we adapted a previously published intertemporal choice task (43) (Figure 1C). The task consisted of 45 trials (decreased from 102 trials in the original task). In each trial, subjects chose between a smaller sooner monetary reward that could be received at end of the current week or a larger monetary reward that was presented with a variable delay. The delayed reward was offered with a one-to-three-week delay and was always at least $0.50 larger than the immediate reward. The delayed reward was presented in the following increments: for $0.20 immediate offers, delayed rewards were $0.70, $1.20, $2.20, $4.20, and $6.60; for $0.50 immediate offers, delayed rewards were $1.00, $1.50, $2.50, $4.50, and $6.50; and for $1.50 immediate offers, delayed rewards were $2.00, $2.50, $3.50, $5.50, and $6.50. As with the risk task, subjects were told their weekly bonuses reflected their actual choices on this task and were therefore incentivized to complete the task according to their true time preferences (see section “Procedure”).

Trial-by-trial choice data were fit separately for each study day to obtain individual subject and day estimates of discount rates (κ). We modeled choice data using a linear utility hyperbolic discounting model (44) (Eq. 3). The utility (*U*) of each option in each trial was calculated from the amount of money offered (*v*), the delay (*d*) to the delivery of *v* where the immediate option was set to a delay of 0 or 7 days (see below), and a subject-specific discount rate (κ).


$$U_{o\,p\,t\,i\,o\,n}=\frac{v}{1+\kappa \,d}$$

To estimate κ, we fit a logistic function (Eq. 4) to the trial-by-trial choice data using a lower and upper bound of [0.0001,1] for κ. The probability of choosing the delayed option (*Pr*_*delayed*_) was derived from the utilities (obtained from Eq. 3) of the immediate (*U*_*immediate*_) and delayed (*U*_*delayed*_) options, and a second subject-specific choice stochasticity parameter (γ).


$$P\,r_{d\,e\,l\,a\,y\,e\,d}=\frac{1}{1+e^{-\gamma \,\left(U_{d\,e\,l\,a\,y\,e\,d}-U_{i\,m\,m\,e\,d\,i\,a\,t\,e}\right)}}$$

Since subjects received their bonuses weekly and potentially could have received their bonus between 1 and 7 days from any given study day (if they selected the “this week”/immediate option), we compared models where (1) the immediate option was 0 days and the delays were 7, 14, and 21 days; and (2) the immediate option was 7 days (the maximum time for an immediate reward) and the delays were 14, 21, and 28 days. We calculated Bayesian Information Criterion (BIC) scores for each model, subject, and day (see Supplementary Table 1). We found that across both groups and across study days, the model assuming a delay of 0 days for the immediate option provided an overall better fit, consistent with an as-soon-as-possible-like effect in discounting as previously reported (45). We also tested whether discount rates were influenced by time to bonus payout but did not find any significant “payday” effects [*t*(804.91) = 0.47, *B* = 0.02, 95% CI [−0.08,0.12], *P* = 0.64] or interactions with diagnosis [*t*(803.66) = 1.07, *B* = 0.07, 95% CI [−0.06,0.2], *P* = 0.29]. Therefore, for all analyses, we report discount rates estimated from the 0 days model. Additionally, given known skew in κ, we report natural log-transformed values of κ, where higher log(κ) indicates steeper discounting.

#### Psychological State Measures

Following prior work in treatment-engaged OUD (33–36), we used single-item self-report surveys to probe subjects’ current affective and psychological states (Figure 1D). We collected information on self-reported affective states, interpersonal relationship quality, general craving for substances and food, clinical factors, and substance use. Subjects in the first cohort were prompted 2 times per day and subjects in the second cohort were prompted 3 times per day to rate their psychological state on a scale from 0 to 100 on multiple dimensions. To increase comparability between the study cohorts, and facilitate analyses with the task data, ratings were averaged to obtain a single estimate per day. Although the general prompts sampled a range of psychological dimensions, for the current study we focused on positive and negative mood as a global assessment of affective “state” relevant to all subjects. This was measured by the questions, “Right now how much do you feel in a good mood?” and “Right now how much do you feel sad, unhappy, down in the dumps, or miserable?” from 0 (“not at all”) to 100 (“very much”). As expected, positive and negative mood were strongly anticorrelated with each other. Further, prior work suggests increased positive and decreased negative mood largely reflect similar relationships to economic (e.g., risk) preferences (46). We therefore calculated a composite mood measure by averaging self-reported positive mood and the inverse of self-reported negative mood at each day and used this measure for analyses with decision-making behavior. Confirming composite mood provided a good approximation for subjects’ overall affective state, we found better mood was significantly negatively related to self-reported worry, hopelessness, agitation, anger, and emotional pain and positively related to appreciativeness, energy, and social connectedness (see Supplementary Figure 1). Additional survey domains related to substance use were omitted from consideration in the current study due to low rates of occurrence in the current sample (e.g., illicit opioid use) or because they were outside the study’s scope.

### Procedure

Subjects completed surveys and the decision-making tasks on their own smartphones *via* an EMA app (MetricWire Inc.). Subjects answered EMA prompts at random intervals on a daily basis over the course of 28 days. EMA data were collected for up to 7 times per day in the first cohort (the first *n* = 22 enrolled subjects) and up to 5 times per day in the second cohort (the remaining *n* = 22 subjects). The first assessment (morning prompt) was collected at 9AM and captured sleep, general outlook for the day, recent drug use, and treatment adherence. General survey questions (∼30 questions each) were administered randomly between 9AM and 9PM, twice per day in the first cohort and alongside the task prompts in the second cohort. These general questions captured affective, psychological, and clinical self-reported states, as described above. At 9PM, the final prompt (night prompts) captured global assessments of the past day, and drug use that was not measured from the earlier prompts. In the first cohort, the tasks and general surveys were randomly prompted at separate times between 9AM and 9PM. To increase co-compliance and reduce burden by reducing the total number of data collection time points within a day, in the second cohort, tasks and general prompts were triggered concurrently between 9AM and 9PM. Subjects in both cohorts had 2 h before prompts expired. Each prompt, including the tasks, took approximately 2–5 min to complete. If subjects failed to start their assigned prompts within 15 min, they received a reminder notification through the MetricWire app.

Prompts and tasks were incentivized to increase compliance and responding according to subjects’ true risk and time preferences. In the first cohort, subjects received $0.50 for each self-report survey completed (e.g., morning, night, and two general prompts), up to $2.00 per day. In the second cohort, subjects received $0.50 per prompt completed, including task prompts, up to $3.50 per day. Subjects in both cohorts received a variable bonus (paid out weekly) with bonuses calculated from randomly selecting one trial from one task prompt from each day (risk in gains, risk in losses, and intertemporal choice). The choice made on the selected trial determined the bonus amount, up to $6.60 per day. Bonuses obtained from a “gain” trial reflected a subject’s real choice and were either $0.50 (if the guaranteed option was selected) or the lottery outcome ($0 or *v*), played according to its probability. If a “loss” trial was selected for realization, $0.50 or the outcome of the lottery ($0 or *v*) was subtracted from $6.60, and the remainder was given as bonus. If the selected trial was from the intertemporal choice task, the amount associated with the chosen option was given as bonus ($0.20–$6.60). Due to a bug in our bonus payment mechanism, bonuses were always added to the current week’s payout (as opposed to being received with the appropriate delay, if a delayed option was chosen). However, as the intertemporal choice task only had 1/3 probability or lower of being chosen for bonus payout each day and subjects were unaware of which trials therein were randomly selected, this is unlikely to have significantly influenced choice behavior (and indeed, we did not observe any systematic “payday” effects as reported above). For cohort 2 only, the bonus could also be drawn from a self-report prompt, which was always worth $3.30 if completed. No bonus was received if the subject missed the response (self-report or task prompt). Subjects had the option to be paid *via* Visa gift cards or to receive cash in person at the end of each week, up to 4 times, while participating in the study.

### Data Analysis

To examine the feasibility of the study design, we calculated prompt compliance rates (number of EMA prompts sent vs. number completed) over the entire enrollment period per subject. We compared compliance rates between cohorts 1 and 2, and between patients and controls with independent samples *t-*tests. To examine the relationship across task parameters, and across each parameter with itself over time, pairwise correlations were run across all subjects for each study day. To directly quantify between- vs. within-person contributions to task parameter variation, we constructed unconditional linear mixed effects (LME) models predicting day-to-day variation in each parameter (max 28/person) from only a subject-specific random intercept. From this model, the intraclass correlation coefficient (ICC) was computed using *EMATools* (47) in R (48). These analyses were performed in the native resolution of the data, as well as in down-sampled (week-level) data as described in Results. To assess for group (aggregate-level) differences between patients and controls, diagnosis was added to LME model. Finally, to determine the degree to which variation in task parameters could be explained by within-person vs. between-person differences in global affective state, participant-mean centered (i.e., within-cluster centering) and participant-mean levels of global mood were added as predictors, as well as their interaction with diagnosis. To allow for comparability between cohorts and to align these data with task data, prompts that probed for psychological state were averaged to derive a single estimate per day for each subject (up to 2 general prompts/day in the first cohort and up to 3 general prompts/day in the second cohort). LME models were estimated using *fitlme* in MATLAB and included a fixed effect for task repetition (number of times a subject had completed a given task up to and including the current study day). Degrees of freedom for significance testing were computed using Satterthwaite approximation. Missing data were not imputed and were censored in analyses.

## Results

### Compliance

**FIGURE 2:**
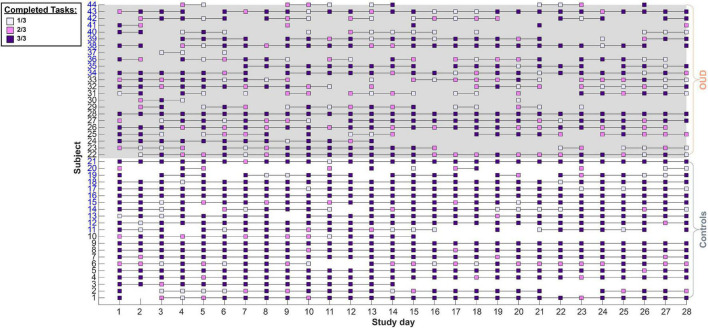
Study compliance with task prompts. Subjects completed up to three decision-making tasks daily. Each square denotes at least one completed task per day. Lines connect successive days that a subject had completed at least one task. Subject numbers on the y axis in black denote cohort 1 while blue denotes cohort 2. In cohort 1, the three tasks (risk in gains, risk in losses, and intertemporal choice) were administered as three separate prompts while in cohort 2 these were administered as two prompts (grouping the gain and loss risk tasks in one prompt). Overall, subjects maintained a moderate (66.88%) compliance rate with controls tending to be more compliant than patients (see section “Results”).

Subjects completed a total of 4603 unique prompts (57.48% of all possible prompts, *M* = 104.61 per person, *SD* = 37.98) over 1085 unique days (*M* = 24.66 person days, *SD* = 5.84). There were no significant differences in the number of unique prompts completed between subjects enrolled as part of cohort 1 (*M* = 103.50 per person, *SD* = 37.80) vs. cohort 2 [*M* = 105.73 per person, *SD* = 39.97; *t*(42) = −0.19, *P* = 0.85]. However, healthy controls completed more prompts (*M* = 124.67 per person, *SD* = 28.15) over more days (*M* = 26.43 person days, *SD* = 4.04) relative to the number of unique prompts completed by patients with OUD [*M* = 86.30 per person, *SD* = 36.95 over *M* = 23.04 person days, *SD* = 6.79; *t*_prompts_(42) = 3.85, *P* = 0.0004; *t*_days_(42) = 1.98, *P* = 0.05]. Task-specific compliance was moderate at 66.88% across subjects (out of three possible tasks per day; Figure 2), with healthy controls (*M* = 80.84%, *SD* = 21.27) being more compliant than patients with OUD [*M* = 54.14%, *SD* = 26.11; *t*(42) = 3.70, *P* = 0.0006]. Given that a small number of patients with OUD (*n* = 4) had especially low task compliance rates (*M* = 13%, *SD* = 0.09, <23% or ∼2 standard deviations lower than the overall sample), we examined what impact that might have on our conclusions. Despite a 16% increase in estimated compliance when removing these *n* = 4 subjects, compliance rates in OUD patients remained below those in controls [*t*(38) = 2.81, *P* = 0.008] and we found no appreciable differences between analyses including vs. excluding these subjects. Therefore, the analyses reported include the full sample.

### Task Parameter Independence and Variability Across Days

**FIGURE 3:**
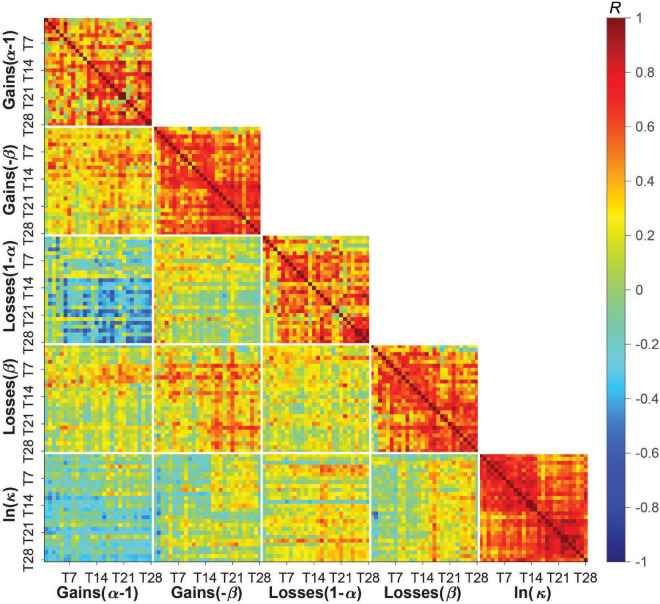
Pairwise correlations between and within task parameters across all study days and subjects. Subjects’ risk, ambiguity, and discounting parameters were only weakly to moderately correlated with each other across subjects and days (blue/green tiles away from the identity line) but moderately to strongly correlated with themselves across study days (red tiles near the identity line). Low to moderate correlations between parameters indicate some degree of independence across these measures of a person’s decision-making profile. Moderate to high correlations within parameters (i.e., day-to-day correlations) suggests reasonable test-retest reliability, which was also measured by the intraclass correlation coefficient (see section “Results”) and found to differ somewhat across parameters (with discounting having the highest reliability/lowest within-person variability). Color bar shows Pearson’s *R* [–1,1].

Figure 3 shows pairwise correlations for each task parameter across all study days and subjects. Comparing decision-making preferences across the gain-loss condition frame and across days, we found risk tolerance in gains was only weakly correlated with risk tolerance in losses (mean *R* = −0.16, *SD* = 0.18). This relationship became stronger and more negative over time. We also observed a moderate correlation between ambiguity tolerance in gains and ambiguity tolerance in losses that similarly became stronger over time (mean *R* = 0.30, *SD* = 0.21). As previously observed in laboratory studies (22, 37–41), risk tolerance in gains was moderately correlated with ambiguity tolerance in gains (mean *R* = 0.32, *SD* = 0.17), and risk tolerance in losses was weakly correlated with ambiguity tolerance in losses (mean *R* = 0.19, *SD* = 0.16). Interestingly, these relationships were maintained over time. Finally, discounting was only weakly correlated with all other parameters (range in mean *R* of |0.05–0.20|). A similar pattern was observed when we averaged behavior across time per subject (Supplementary Figure 2). Collectively, these data indicate that the different task parameters assessed captured at least partly distinct aspects of subjects’ value-based decision-making behavior.

By contrast, the task parameters were moderately to highly correlated with themselves over time, suggesting a degree of stability in these preferences over the 28 days. The average correlation over time for ambiguity tolerance was higher than for risk tolerance in both gains (ambiguity: *M* = 0.57, *SD* = 0.20; risk: *M* = 0.45, *SD* = 0.26) and losses (ambiguity: *M* = 0.53, *SD* = 0.23; risk: *M* = 0.44, *SD* = 0.25), and highest for delay discounting (*M* = 0.65, *SD* = 0.16).

We formally quantified how much of the variation in task parameters was due to between-person (i.e., person-to-person) vs. within-person (i.e., day-to-day) variability using ICC. Roughly 47–60% of the variation in risk and ambiguity tolerance, and 33% of the variation in delay discounting, could be attributed to within-person variation. Risk tolerance in losses had the lowest ICC and was thus most influenced by within-person factors (ICC = 0.40), followed by risk tolerance in gains (ICC = 0.43), and ambiguity tolerance in gains and losses (ICC = 0.53 for both), with discounting showing more between-person than within-person variation (ICC = 0.67). Further, within-person variation was higher in patients than controls for all parameters (patients: ICC = 0.37, 0.49, 0.43, and 0.61, controls: ICC = 0.42, 0.56, 0.60, and 0.65 for risk tolerance in losses, ambiguity tolerance in gains, ambiguity tolerance in losses, and discounting, respectively) except risk tolerance in gains (patients: ICC = 0.49, controls: ICC = 0.40).

In our prior lab-based study (22), we examined risk tolerance and ambiguity tolerance in gains at the roughly week-to-week level. For comparison to our current findings at the day-to-day level, we down-sampled subjects’ 28 days of data to 4 timepoints by averaging all parameters provided by each subject in a given week. We observed a marked reduction in within-person variability for all parameters. At the week-level, roughly 23–37% of the variation in risk and ambiguity tolerance, and 22% of the variation in delay discounting, could be attributed to within-person variation, with ICCs ranging from 0.63 to 0.77. To confirm that this reduction was not explained simply by reduced error due to averaging, we instead down-sampled the data by randomly selecting days from each week that were at least 4 days apart which is comparable to randomly testing individuals in the lab. We observed similar reduced variability across parameters with 26–47% of the variance explained by within-person variation (ICCs ranging from 0.53 to 0.74). Overall, this suggests that risk and time preferences, at least as assessed “in the wild,” are more variable than originally considered and may require more frequent sampling (e.g., days vs. weeks) to capture their full dynamic range.

Finally, we tested for linear trends over time in task behavior that depended on task repetition (number of times a subject had completed a given task up to and including the current day) and its interaction with diagnosis. We found a linear fixed effect of repetition for risk tolerance in losses [*t*(806.64) = −4.19, *B* = −0.08, 95% CI [−0.12,−0.05], *P* = 3.05*10^–5^], ambiguity tolerance in losses [*t*(797.01) = −3.04, *B* = −0.06, 95% CI [−0.10,−0.02], *P* = 0.002], and discounting [*t*(818.86) = −3.56, *B* = −0.06, 95% CI [−0.10,−0.03], *P* = 3.93*10^–4^]. With time, subjects became less tolerant of risk and ambiguity in losses and more patient (Supplementary Figure 3). This effect for ambiguity tolerance in losses was stronger in patients than controls [*t*(792.86) = −3.04, *B* = 0.05, 95% CI [0.005,0.12], *P* = 0.10; all other interaction effects, *P* > 0.08]. There were no significant repetition effects for risk [*t*(798.11) = −0.27, *B* = −0.005, 95% CI [−0.04,0.03], *P* = 0.79] nor ambiguity tolerance [*t*(790.82) = 0.44, *B* = 0.008, 95% CI [−0.03,0.04], *P* = 0.66] in gains.

### Exploring Potential Sources of Variability: Diagnostic Group Differences and Relationship to Fluctuating Psychological State

**FIGURE 4:**
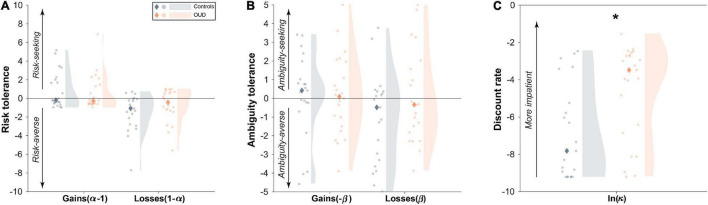
Diagnostic group differences in aggregate level decision-making parameters. **(A)** Aggregate-level risk tolerance and **(B)** ambiguity tolerance in both gains and losses were comparable between people with opioid use disorder (OUD) and matched controls. By contrast, **(C)** patients had higher discount rates overall than controls. Dots represent individual subject averaged data across the 28 study days. Shaded regions represent a density plot of subjects’ averaged risk and time preferences. Diamonds indicate group medians. *P = 0.006.

In exploratory analyses, we assessed for potential sources of variability in risk and time preferences. Like lab-based findings, subjects were sensitive to risk, ambiguity, and delay, favoring more certain (especially in losses) and more immediate rewards (Figure 4). Given the theoretical impetus for aggregate-level differences in addiction, we examined as a potential source of *between-person* variation diagnostic group differences. Overall, patients had significantly higher discount rates indicating more impatient choices relative to controls [controlling for task repetition, *t*(44.23) = −2.88, *B* = −2.17, 95% CI [−3.69,−0.65], *P* = 0.006]. However, there were no significant aggregate-level group differences for risk tolerance in gains [*t*(44.01) = 0.48, *B* = 0.28, 95% CI [−0.89,1.44], *P* = 0.63] or in losses [*t*(44.57) = −1.40, *B* = −0.80, 95% CI [−1.96,0.35], *P* = 0.17], or for ambiguity tolerance in gains [*t*(43.33) = −0.03, *B* = −0.02, 95% CI [−1.34,1.30], *P* = 0.97] or in losses [*t*(42.35) = 2.56, *B* = −1.11, 95% CI [−2.56,0.34], *P* = 0.13].

**FIGURE 5:**
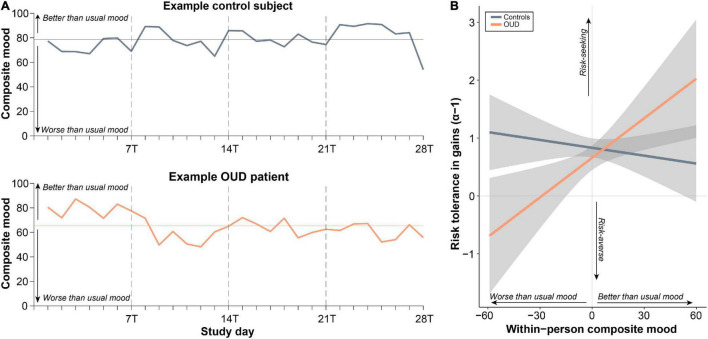
Within-person changes in psychological mood state relate to changes to risk tolerance in gains. **(A)** Ratings of composite mood showed substantial within-person variability across the study days as determined by the intraclass correlation coefficient (ICC; variance due to between-person relative to within-person variability). **(B)** Better than usual mood was associated with more risk tolerance, especially in patients with opioid use disorder (OUD) (see section “Results”).

We also examined the relationship between fluctuating mood state and the task parameters as a potential source of *within-person* variation. As expected, global mood varied across days (Figure 5A), with 47% of this variability attributed to within-person (vs. between-person) variation (ICC = 0.53). Unlike for task parameters, ICCs were higher for patients compared to controls (patients: ICC = 0.59, controls: ICC = 0.49). However, we found that on *days* of better than usual reported mood, subjects were more risk tolerant in gains [*t*(746.92) = 2.44, *B* = 0.02, 95% CI [0.004,0.04], *P* = 0.015]. This relationship tended to be stronger in patients as demonstrated by a group × person-mean centered composite mood interaction [*t*(746.31) = −2.41, *B* = −0.03, 95% CI [−0.05,−0.005], *P* = 0.016; Figure 5B]. Adding to the model task repetition did not impact these effects (*P* < 0.02), suggesting independent influences on task behavior. There was no significant between-person effect of mood such that risk tolerance in gains was not higher in subjects who reported overall better mood (main effect: *P* > 0.18, interaction with diagnosis: *P* > 0.77). Global mood also did not track at the within- or between-person levels with any of the other task parameters (main effects: *P* > 0.28, interactions with diagnosis: *P* > 0.15).

## Discussion

People with drug addiction are thought to engage in risky and impulsive behaviors despite harmful consequences related to drug use, suggesting that addiction may affect cognitive processes related to decision-making about uncertain and delayed rewards. While addiction is considered a dynamic and multifaceted disorder, research examining decision-making remains largely cross-sectional and typically only focuses on a single feature of this behavior at a time. In this pilot study, we leveraged a smartphone-based ecological momentary assessment platform to determine the feasibility of capturing day-level variation in risk and time preferences and linking these to changes in psychological state in people with chronic OUD.

Our preliminary results suggest that probing cognitive decision-making processes with behavioral tasks in patients through EMA is feasible for capturing the temporal dynamics of risk and time preferences “in the wild.” Compliance rates were moderate and somewhat lower compared to other EMA studies (49) using survey (33, 34, 36, 50) and task (23, 24) prompts. However, compliance for task and general survey prompts were still acceptable given that most studies have not attempted to probe multiple cognitive processes at a time and with high temporal resolution, especially in a patient sample. We also found after excluding individuals with low compliance, our findings did not change, suggesting that compliance is unlikely to significantly impact our conclusions. In our first cohort, we observed that compliance was greater for task prompts vs. survey prompts, which could be attributed to the larger incentives received compared to other survey prompts. We attempted to mitigate this in the second cohort by increasing incentives across all prompts with positive results, which stresses the importance of incentives for increasing compliance. Higher compliance was also noted in healthy controls as these individuals may not have experienced the pressures related to socioeconomic hardship as much or unpleasant symptoms related to OUD. Treatment adherence may have some influence on compliance in patients, however, given that other EMA studies report little to no effect of treatment status (49) and the low rates of illicit drug use in our sample, this relationship is also likely to be weak. Future work is necessary to examine how subject-level factors and other incentives (e.g., gamification) can offset low compliance.

Consistent with previous literature, risk and ambiguity tolerance and discounting did not correlate strongly with each other (22, 37, 41, 38, 39) but did demonstrate acceptable test-retest reliability (22, 51, 52). This suggests that these preferences uniquely contribute to a person’s decision-making profile and may be important to examine together over time (20). We found that risk tolerance and ambiguity tolerance were more variable across days than delay discounting, with patients demonstrating more variability overall than healthy controls. Some of the variation in risk tolerance was explained by within-person changes in mood state, especially in OUD. By contrast, discounting was significantly higher in aggregate in OUD compared to controls but unrelated to global mood state. This finding supports discounting as having a stable, robust relationship to addiction (53–58) that may be clinically relevant in the development and maintenance of the disorder, while taken together with our prior study (22), risk and ambiguity tolerance might more closely reflect the current state of the person.

Day-level estimates of ICC were lower than what we previously found for a subset of the parameters at the week-to-week level [i.e., risk and ambiguity tolerance in gains (22)]. This may have been attributed to the increased frequency of data collection as there may be more variability day-to-day vs. week-to-week. We confirmed this by recomputing ICCs for: (1) parameters averaged across days within each week; and (2) randomly sampled days from each week. In these down-sampled data, we found higher ICCs suggesting differences could be in part explained by increased day-level variation. Alternatively, increased within-person variability could be attributed to external influences outside of normal laboratory settings such as additional distraction in remote settings. However, given relatively high ICCs for discounting in both day-level and week-level data, this is less likely. Taken together, these data suggest that at least a week-level (and likely day-level) sampling frequency may be needed to capture meaningful within-person changes in addiction (36).

We found that our sample was sensitive to risk and delay, favoring certain (especially in losses) and immediate rewards over uncertain and delayed rewards, consistent with our prior findings (22). As in that study, we did not find aggregate-level group differences in ambiguity tolerance. Unexpectedly, however, we also did not find aggregate-level group differences between patients and controls in risk tolerance. This may be attributed to differences in sample characteristics such as the older age, greater proportion of females, and lower rates of illicit drug use in our current sample, and our small sample size which may only be sensitive to detect larger effect size differences. By contrast, consistent with an extensive prior literature showing large effect size group differences (7, 9), we found that patients with OUD displayed steeper discounting than healthy controls. Interestingly, this difference was observed even with immediate rewards that were not truly immediate. In our task, the “immediate” rewards could have been received with a 1–7-day delay, overall revealing that individuals with OUD may consider delayed rewards with respect to the “as-soonest-as-possible” reward (45) while engaging in their natural environments.

Decision-making is thought to be a process sensitive to within-person changes such as mood state (26, 59). Therefore, we preliminarily examined the variability of psychological state, measured by positive and negative mood, and tested whether these state changes map onto changes in risk and time preferences. We found that on days of better than usual mood, subjects tended to be more risk tolerant in gains, with this effect tending to be stronger in patients, consistent with prior pharmacological and functional neuroimaging findings suggesting a role for dopaminergic function in both elevated mood and risk-taking behavior (60–62). This implies that within-person changes in global indices of affect may influence risk tolerance which may be consequently related to changes in broader risk-taking behavior (beyond drug use). We did not observe, however, any additional relationships between psychological state and the other decision-making parameters nor did we find differences between individuals who generally reported better moods. While ambiguity tolerance and time preferences were unrelated to global indices of mood state, it is possible that they could track with other dynamic variables not assessed here (e.g., clinical state, social context, or prior beliefs) (22, 63–66).

## Limitations

This proof-of-concept study was optimized for high-frequency probes of decision-making preferences which ultimately led to a smaller sample size. The trade-off was reduced power to detect smaller effect size group-level differences. Given that we only enrolled subjects with access to internet-enabled smartphones, we may have only captured those with less severe symptom profiles, and conversely, observed low rates of illicit drug use and clinically relevant changes (e.g., craving). We were thus unable to link changes in decision-making preferences to risk for reuse/relapse as in our previous work. While a strength of EMA is the ability to capture decision-making preferences in the subject’s natural environment, this method may be susceptible to external influences such as increased distractibility. These external factors may lead to increased noise that is not readily distinguishable from meaningful within-person variability. Additionally, the dense repeated measures design of EMA may increase the likelihood of repetition effect, as we indeed observed. While the repeated nature of the study allows for building rapport with subjects, establishing a level of trust that they would receive their payments, this and cumulative earnings could have influenced economic decisions in our sample in a time-dependent manner. Lastly, patients were recruited during the COVID-19 pandemic from two separate treatment programs which may have had additional unknown effects on behaviors.

### Conclusion and Future Directions

In summary, to our knowledge, the current pilot study is the among the first to use EMA to probe economic risk and time preferences simultaneously in people with drug addiction. We demonstrated that using EMA to capture cognitive processes in patients with OUD is feasible and may be crucial to capturing dynamic patterns of decision-making. Preliminary findings suggest that decision-making preferences, such as risk tolerance, are dynamic at the day-to-day level and may be sensitive to within-person changes in psychological state. Future work will be necessary to further discern the influence of other state variables, such as clinical state, on changes in decision-making preferences. These preliminary findings support the utility of studying decision-making in more ecologically valid settings and which may reveal temporally relevant treatment targets for addiction.

## Data Availability Statement

The data supporting the conclusions of this article will be made available by the authors on reasonable request.

## Ethics Statement

The studies involving human participants were reviewed and approved by the Rutgers University Institutional Review Board. The patients/participants provided their written informed consent to participate in this study.

## Author Contributions

EA and AK designed the research with input from EK. SH and DB collected the data. EA and AK analyzed the data and wrote the first draft of the manuscript. All authors provided comments on the final version.

## Conflict of Interest

The authors declare that the research was conducted in the absence of any commercial or financial relationships that could be construed as a potential conflict of interest.

## Publisher’s Note

All claims expressed in this article are solely those of the authors and do not necessarily represent those of their affiliated organizations, or those of the publisher, the editors and the reviewers. Any product that may be evaluated in this article, or claim that may be made by its manufacturer, is not guaranteed or endorsed by the publisher.
