# Supplementary material for: A Proof-of-Concept Ecological Momentary Assessment Study of Day-Level Dynamics in Value-Based Decision-Making in Opioid Addiction
Source: Front Psychiatry. 2022 May 17;13:817979. doi: 10.3389/fpsyt.2022.817979 (PMC9156899; doi:10.3389/fpsyt.2022.817979)
Supplement: Supplementary file 1 [file Data_Sheet_1.pdf]

Supplementary Material

1. Supplementary Figures and Tables

1.1 Supplementary Figures

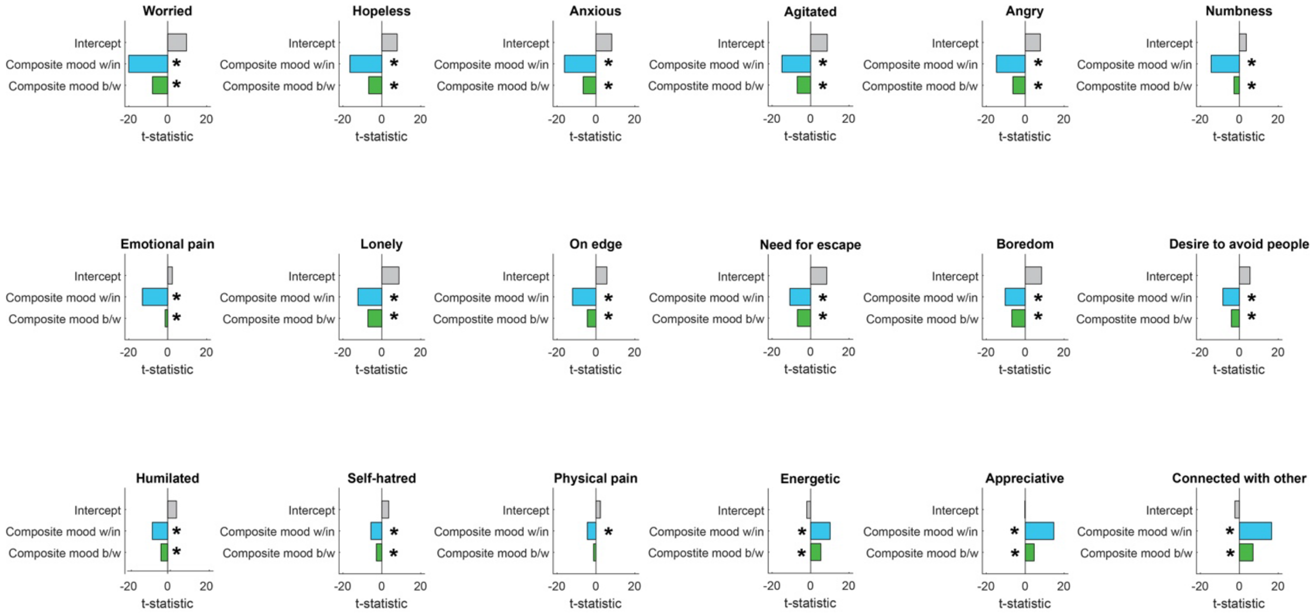

Supplementary Figure 1. *Relationship of within-person change and between-person differences in composite mood with other psychological state variables measured concomitantly.* Better mood at either the within- or between-person levels was significantly negatively correlated with feelings of worry, hopelessness, agitation, anger, and emotional pain and positively correlated with feelings of appreciativeness and connectedness with others. W/in: within. B/w: between. * *P*<0.05

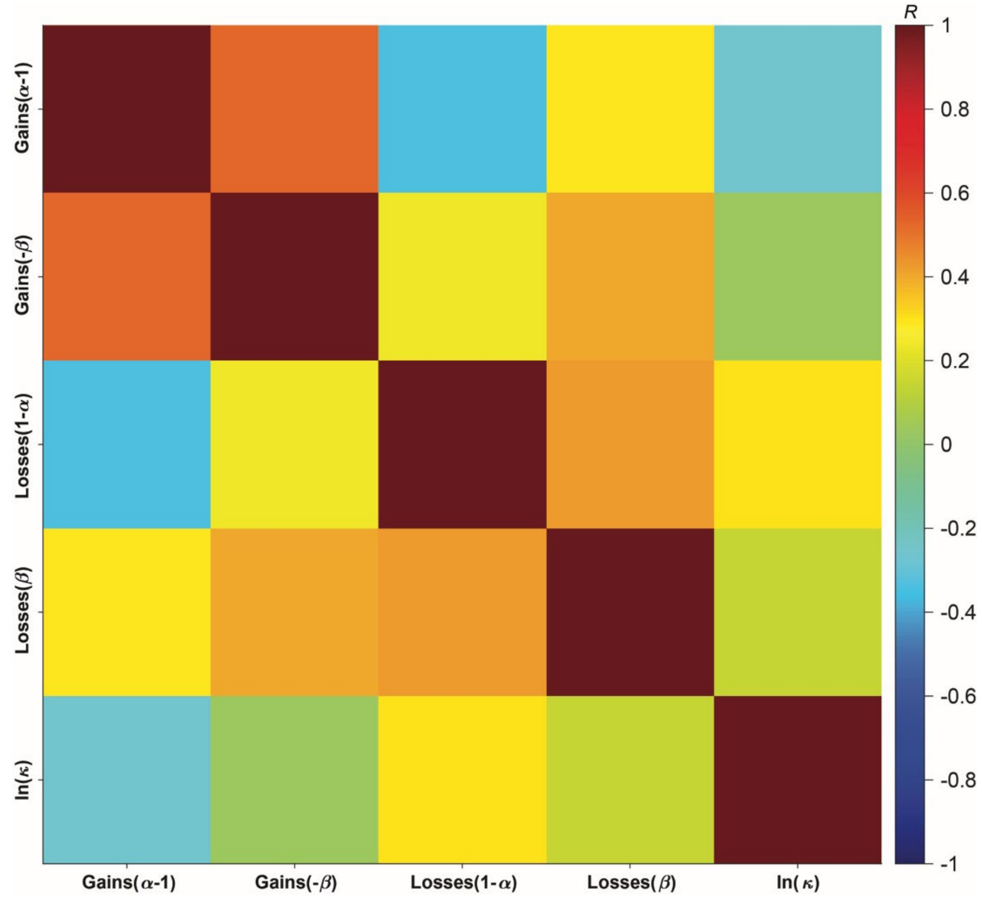

Supplementary Figure 2. *Pairwise correlations between and within task parameters averaged across all study days per subject*. Color bar shows Pearson's *R* [-1,1]).

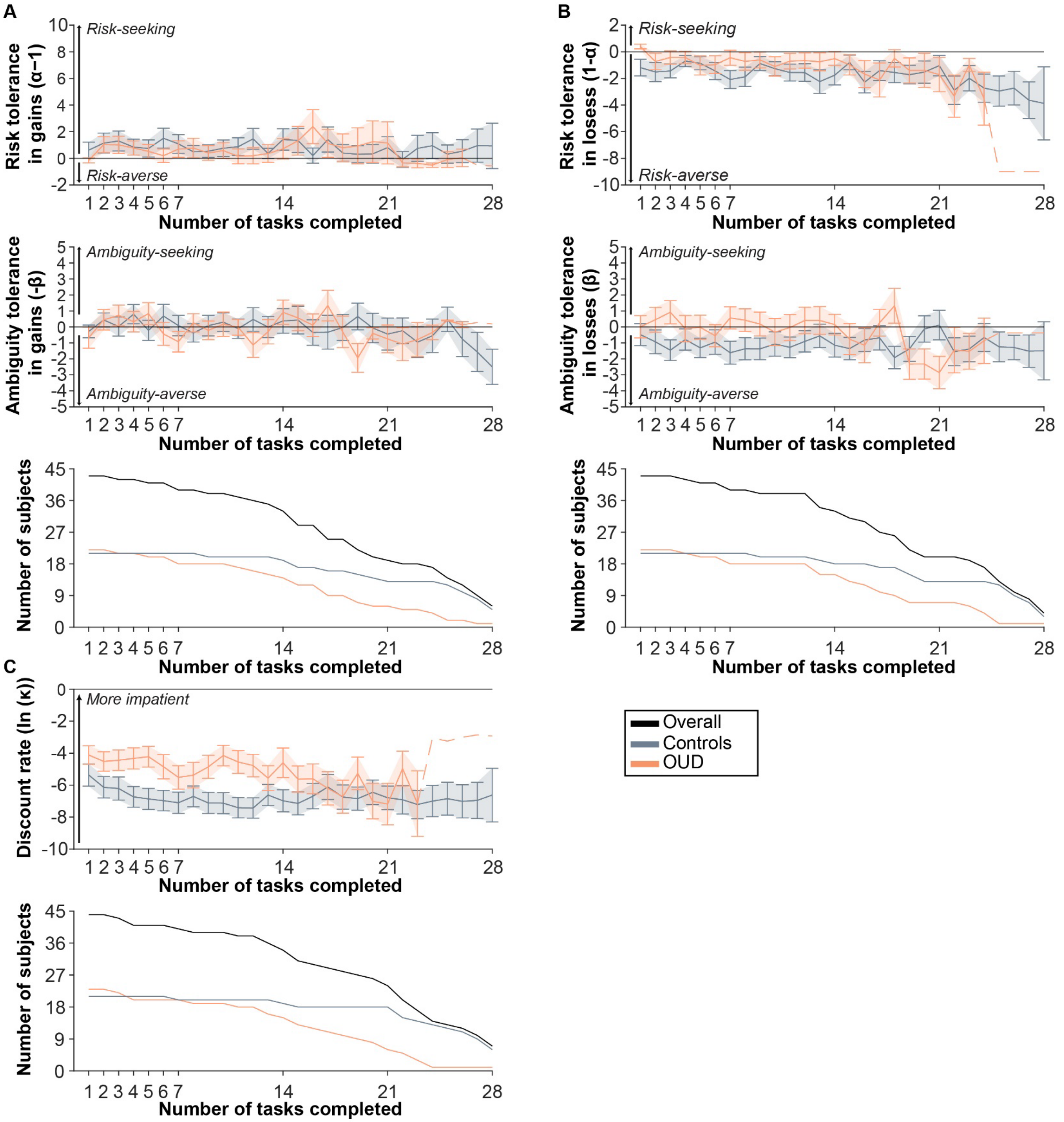

Supplementary Figure 3. *Relationship between the number of times subjects completed a given task and estimated decision-making parameters per group (repetition effects). Dotted lines represent a single subject.*

1.2 Supplementary Tables

	Overall		Controls		OUD	
	r^2	BIC	r^2	BIC	r^2	BIC
**Intertemporal choice task**						
Immediate option, delay=0 days	0.759	22.649	0.835	17.887	0.689	26.996
Immediate option, delay=7 days	0.745	23.537	0.827	18.395	0.669	28.231
**Risk task (gains)**	0.569	44.200	0.613	40.920	0.528	47.331
**Risk task (losses)**	0.563	44.707	0.596	42.186	0.530	47.115

Supplementary Table 1. *Model fit indices for each parameter and task.* Average model fit indices (BIC, Bayesian information criterion, and adjusted-r^2) in the overall sample, controls, and individuals with opioid use disorder (OUD). The immediate delay option = 0 days model fit better than the immediate delay option = 0 days across all subjects and all days.
